# Strong anti-viral nano biocide based on Ag/ZnO modified by amodiaquine as an antibacterial and antiviral composite

**DOI:** 10.1038/s41598-022-24540-8

**Published:** 2022-11-19

**Authors:** Mahboubeh Dolatyari, Ali Rostami

**Affiliations:** 1 Industrial Park of Advanced Technologies, ASEPE Company, Tabriz, 5364196795 Iran; 2 Photonics and Nanocrystal Research Lab (PNRL), Faculty of Electrical and Computer Engineering, University of Tabriz, Tabriz, 5166614761 Iran

**Keywords:** Biochemistry, Health care, Chemistry, Materials science, Nanoscience and technology

## Abstract

In this paper, we synthesized Ag/ZnO composite colloidal nanoparticles and the surface of nanoparticles was improved by amodiaquine ligand. The synthesized nanoparticles were characterized using the XRD diffraction pattern, FT-IR Spectroscopy, TEM image, and UV–Vis spectroscopy. The antibacterial, antifungal, and antiviral effects of the synthesized colloid were examined on *E.coli, Staphylococcus aureus, Pseudomonas aeruginosa,* and *Enterococcus hirae bacteria,* and *Candida Albicans* and *form spore aspergillus* fungi*,* also *influenza, herpes simplex,* and *covid 19* viruses. The results indicate more than 7 log removal of the bacteria, fungi, and viruses by synthesized colloid with a concentration of 15 μg/L (Ag)/50 µg/ml (ZnO). This removal for *covid 19* virus is from 3.2 × 10^8^ numbers to 21 viruses within 30 s. Also, irritation and toxicity tests of the synthesized colloid show harmless effects on human cells and tissues. These colloidal nanoparticles were used as mouthwash solution and their clinical tests were done on 500 people infected by the coronavirus. The results indicate that by washing their mouth and nose three times on day all patients got healthy at different times depending on the depth of the disease. Almost all people with no signs of infection and using this solution as a mouthwash didn’t infect by the virus during the study.

## Introduction

Complications and types of resistant and unknown viruses have made viral infections a major global health challenge. Due to the complexity of virus behavior, long-term use of antivirals reduces the usefulness of treatment for pathogenic viruses^1–6^. According to the World Health Organization (WHO), humans have been exposed to several viral infections since the beginning of the twenty-first century. Acute Respiratory Syndrome of *Coronavirus* (*SARS-CoV*), *H1N1 Influenza*, and Coronavirus Respiratory Syndrome of the *Middle East (MERS-CoV)* started in 2002, 2009, and 2012 in different countries respectively. Recently, another viral epidemic occurred called “*COVID-19*” or “Coronavirus 2019”. As it is highly contagious, the new virus has affected human life, the global economy, and people's livelihoods^7–9^. Many scientists and pharmaceutical companies have done extensive research over the past two decades to discover an effective and organized way to protect human lives against infectious diseases caused by the coronavirus, such as *SARS* and *Mers*. Among these studies, small molecules, ordinary antiviral drugs, and antibody-based drugs are promising for the treatment of viral infections^10–14^. In the case of new viral infections, the development of an effective drug requires a great deal of study, cost, and time. Therefore, in the absence of effective treatment protocols and treatments, virus prevention is one of the best ways to reduce viral infections^15^. Virus prevention can be done in different ways. In the case of respiratory-related viral infections, maintaining a safe distance and removing and/or neutralizing virus particles from the level reduces the risk of infection^16^.

According to the capabilities of the new science and technology, nanotechnology offers a powerful tool for diagnosing, preventing, and treating infectious diseases caused by viruses^10,17,18^. The dimensions of viruses are usually in the nanoscale range, and therefore, the field of nanomedicine investigates the uptake of nanoparticles in the cell and examines the approaches and mechanisms of action of nanoparticles within the cell^19–21^.

Metal, metal oxide, and sulfide nanoparticles have exhibited promising antimicrobial and antiviral activity depending on various parameters such as nature, size, surface area, crystallinity, capping and stabilizer materials, morphology, concentration, pH, and the nature of the microorganism. Smaller particles with suitable morphology can easily penetrate through the nanopores of microorganisms. Therefore, optimizing these parameters can develop new nanomaterials suitable for the treatment of various diseases^22–27^. Various studies have reported the antibacterial properties of different metals and metal oxide and sulfide nanoparticles. Tin ferrite decorated on Bismuth ferrite, Ag/g–C_3_N_4_/SiC**,** gold/iron doped silver iodide**,** Co-doping silver and iron on graphitic carbon nitride**,** Sn/Fe**,** CuO nanorods and CuWO_4_ nanoparticles**,** Ag doped Sn_3_O_4_**,** silver Ferrite/Bismuth ferrite**,** Ag decorated CoO, CuO loaded ZnS nanoflower, Al_2_O_3_ decorated 2D-CdO nano-heterojunction**,** CdS–Ag_2_S, MgS/Ag_2_MoO_4_, Spinel FeV_2_O_4_ coupling on nanocube-like Bi_2_O_3,_ ZnFe_2_O_4_ decorated CdO nanohybrid are samples of these research works^28–44^.

The use of gold nanoparticles to detect viral infections has promising results^45–47^. Silver nanoparticles (Ag NPs) have also been identified as a useful new approach to resistant viral and bacterial strains^10,48,49^ Ag NPs can play an important role in the control and treatment of unknown infectious diseases. Silver nanoparticles (NPs), as one of the most familiar nanoparticles, have been widely used to identify, neutralize and treat viral infections. Silver NPs can be easily synthesized by various methods such as green (biological), chemical and physical. However, there are concerns about the potentially toxic effects of high concentrations of silver-based substances, and studies have been conducted that recommend not using them in high concentrations^10,50–53^. Therefore, the use of these particles for medical purposes should be done considering their advantages and limitations. Ag NPs have been validated as an antiviral agent in humans against many viruses, including human immunodeficiency virus, hepatitis B virus, herpes simplex virus, respiratory syncytial virus, poliovirus adenovirus, and monkeypox virus. Recently, scientists investigated the possibility of Ag NPs as therapeutic agents against coronavirus and finally suggested their use to prevent infection-related coronaviruses^54,55^. In this case, some studies introduce the most common NPs with antiviral activity against animal and human COV viruses^56–59^. Zinc oxide nanoparticles are believed to be non-toxic, biologically safe, and biocompatible. It is used as drug carriers and fillers in the medical materials and cosmetic industries. Several studies have reported the harmful effects of nanoparticles on living cells, but no toxic effects on eukaryotic cells have been reported for low concentrations of zinc oxide nanoparticles^60^. ZnO nanoparticles have been studied for the development of next-generation nano-antibiotics against pathogenic microorganisms to combat multidrug resistance^61–63^. Zinc oxide nanoparticles have a wide range of antimicrobial activity against microorganisms such as *Escherichia coli, Staphylococcus aureus, Pseudomonas aeruginosa, Bacillus subtilis,* and *bacteriophage M13*^64^. For using both Ag and Zinc oxide antibacterial and antiviral effects, here we synthesized Ag/ZnO composite nanomaterials in which the concentration of Ag nanoparticles is in the optimum range of toxicity. One of the advantages of nanoparticles is the capability of their surface to interact with ligands having functional groups. To improve our synthesized drug, the surface of nanoparticles has interacted with the amodiaquine ligand that can interact with viruses, bacteria, and fungi. According to our knowledge, there is no report on the synthesis and characterization of the Ag/ZnO/ Amodiaquine nanoparticles till now. Amodiaquine has been used clinically as an oral antimalarial medication for more than 60 years. However, effective antiviral dosages are much higher to be useful. In our synthesized nanoparticles the concentration of Amodiaquine is less than 0.1 g/L. As a result, we could significantly reduce the bacteria, fungi, and viruses with a low dosage of the synthesized colloid and it was able to improve patients at the start of the illness period.

## Methods

### Synthesis Ag/ZnO

0.024 g silver acetate, and 0.6 g polyvinyl pyrrolidone (PVP), were dissolved in 450 ml deionized water and stirred for 5 min. Then, 0.1 g sodium borohydride was dissolved in 50 ml deionized water, drop wised inserted into the first solution, and stirred for 30 min. 0.11 g Zinc acetate dehydrate was added to the solution and 0.1 g sodium borohydride was dissolved in 500 ml deionized water and drop wised inserted into the solution again. This means the obtained product has 15 ppm Ag and 50 ppm ZnO nanoparticles.

### Surface engineering with amodiaquine ligands

0.1 g 4-[(7-Chlor-4-chinolyl)-amino]-2-[(diethylamino)-methyl]-phenol (Amodiaquin dihydrochloride Dihydrate) dissolved in 20 ml deionized water and inserted on 80 ml of the synthesized colloidal nanoparticles and stirred for 24 h. The obtained nanoparticles were centrifuged and washed with ethanol and water several times.

### Clinical trials

The obtained pure materials were dispersed in 100 ml of deionized water and used as mouthwash solutions for patients.

### Irritation and toxicity test

Four Hindi pigs have been used to perform the test each time. The back’s right caudal and left cranial area of each tested animal has been treated with the examined substance, while the non-treated left tail and right cranial area of the back have been used as control. Approximately 24 h before the test, the fur was removed from an area approximately 240 cm^2^ wide by clipping and shaving the dorsal and flank zones of the animals. An area of the back, about 6 cm^2^ wide, was designed for the application of the test sample.

25 × 25 mm of the test substance was applied directly to the skin on the cranial site of each Hindi pig. The application sites were covered with a non-occlusive dressing and the wrap of the application sites was with a semi-occlusive bandage. The patches were removed 4 h after the application and repeated skin irritation tests are renewals.

4 ml of the synthesized nanoparticles were taken and then to keep the material on the skin, 1% CMC was added to this suspension and allowed exposure to animals. Four groups for experiments were chosen (25, 50, 75, and 100% body surface minus head). The acute toxicity study of this product was performed dermally through the box with the dimension of 50 × 30 × 40 cm on a standard breeding Hindi pig and white albino rabbits obtained from the Pasteur Institute in Tehran. The synthesized material didn’t introduce death or any other behavior or physical damage to the animals as well as did not change food and water consumption. Histopathological observation in vital organs did not show any abnormal permanent effect.

For the Draize rabbit eye test, 100 μL of the test liquid into the lower conjunctival cul-de-sac. Observations of corneal opacity and area of corneal involvement, conjunctival hyperemia, chemosis, ocular discharges, and iris abnormalities are taken at 1, 24, 48, 72 h, 1 week, and 2 months. The test is performed on white albino rabbits due to their large eyes, well-described anatomy, ease of handling, relatively low cost, and ready availability.

### Bacterial growth inhibition test

Five bacterial strains were tested: *E. coli*, *Staphylococcus aureus, Enterococcus hirae, Pseudomonas aeruginosa,* and *Methicillin-resistant Staphylococcus aureus (MRSA).* All were obtained from MikroBank at the University of Tehran, Iran. The plates including bacteria incubated for 24 h at 37 °C and then the colony of bacteria was counted in each milliliter of solution (CFU/ml) (1.5 × 10^8^ CFU/mL). 0.3 g/l of interfering substance (Bovine albumin) was added to 1 ml of bacteria suspension and stored for 2 min at 20 °C. after this time 8 ml of synthesized colloid was added to it. The neutralizer was selected to be 30 g/l polysorbate, 30 g/l saponin, and 3 g/l lecithin. Negative control was prepared by mixing equal volumes of bacteria and deionized water. All samples were then incubated overnight in a shaking incubator under the same conditions.

### Fungal growth inhibition test

The antifungal tests were done on Candida albicans and Aspergillus fungi. The method is the same as bacteria.

### Viral growth inhibition test

1 ml of interfering substance was added to 1 ml virus suspension with the concentration of 1 × 10^–7^ CFU/mL. 8 ml of the synthesized material with a concentration of 15 μg/L (Ag)/50 µg/ml (ZnO) was added to the solution and kept in a water bath (22 °C) for 30 s. After this time the solution was transferred to an ice bath and then the solution was titrated at 37 °C according to the TCID50/ml standard method.

### Ethics approval and Consent to participate

Herby, we confirm that all experiments were performed in accordance with relevant guidelines and regulations. Toxicity and irritation tests were studied at Cell and animal toxicity laboratory, Faculty of Pharmacy, University of Tehran Medical Sciences, Tehran P.O.Box: 14155/6451 I.R..IRAN. The standards for irritation and toxicity experiments and methods were ES, BN ISO 10993: 10; 2016 and BS EN ISO 10993-11(2009). All experimental protocols were approved by IR-FDA and Research Ethics Committees of Tabriz University of Medical Sciences with Approval ID: IR.TBZMED.REC.1400.179. We confirm that informed consent was obtained from all subjects and/or their legal guardian(s). All methods were carried out by relevant guidelines and regulations. Name of the chemical and methods to anesthetize the animal is reported in detail in the materials and method section. All documents related to irritation and toxicity tests and ethics approval are included as supplementary files. Also, the statements are included in the manuscript. We confirm that all experimental protocols were approved by IR-FDA and Dr. Ostad from the Cell and animal toxicity laboratory, Faculty of Pharmacy, University of Tehran Medical Sciences, Tehran. All methods were carried out in accordance with the following standards: Antibacterial Tests: EN1276, Antifungal Tests: EN1650, Antiviral Tests: EN 105, ISIRI 16676, EN 14476. All mentioned standards have been included in the manuscript and test results are included as supplementary files. Irritation and toxicity methods and materials are attached as supplementary files. Also, Figure Number 6 has been taken by Prof. Ostad at Tehran University of medical sciences, Iran. other pictures taken by Prof. Ostad are attached as supplementary files.

## Results and discussion

### Physical characterization

**Figure 1:**
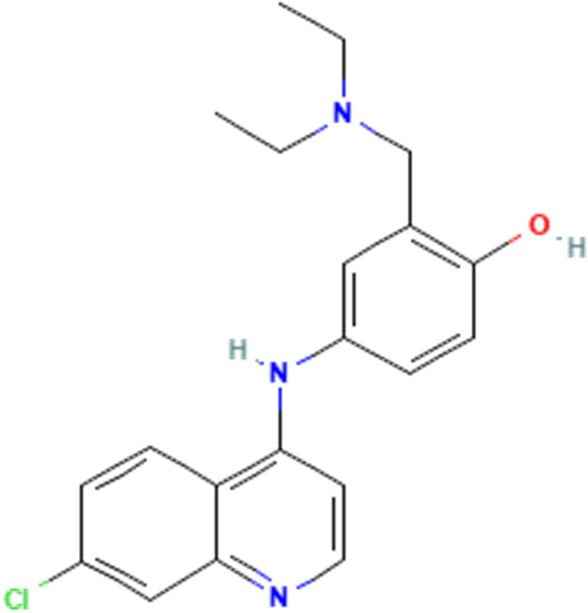
Chemical structure of the amodiaquine compound.

Amodiaquine is an orally active 4-aminoquinoline derivative with antimalarial and anti-inflammatory properties. The molecular Formula of Amodiaquine is C_20_H_22_ClN_3_O and its chemical structure is shown in Fig. 1.

**Figure 2:**
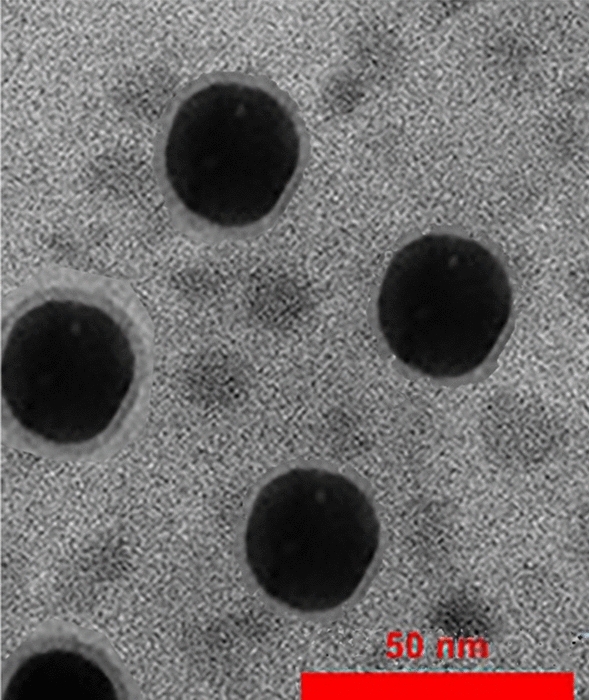
TEM image of synthesized Ag/ZnO/Amodiaquine nanoparticles.

Ag/ZnO nanoparticles were synthesized by the reduction method. In this method, ions Ag^+^ and Zn^2+^ were reduced by sodium borohydride. However, the Zn atoms in the water environment are not stable and convert to ZnO particles. The surface of nanoparticles is covered by PVP in the first step. In the second part, the polymer chains were instituted by an amodiaquine ligand. As Fig. 1 shows, three functional groups make the molecule to be suitable for interaction with the surface of nanoparticles. These groups are –OH, –Cl, and –NH. Figure 2. Shows TEM image of synthesized nanoparticles. As the figure shows there are two types of nanoparticles in the image. Ag nanoparticles are 35 nm and a shell can be observed around the particles. There are ZnO nanoparticles beside the Ag nanoparticles with a diameter of 12.5 nm.

**Figure 3:**
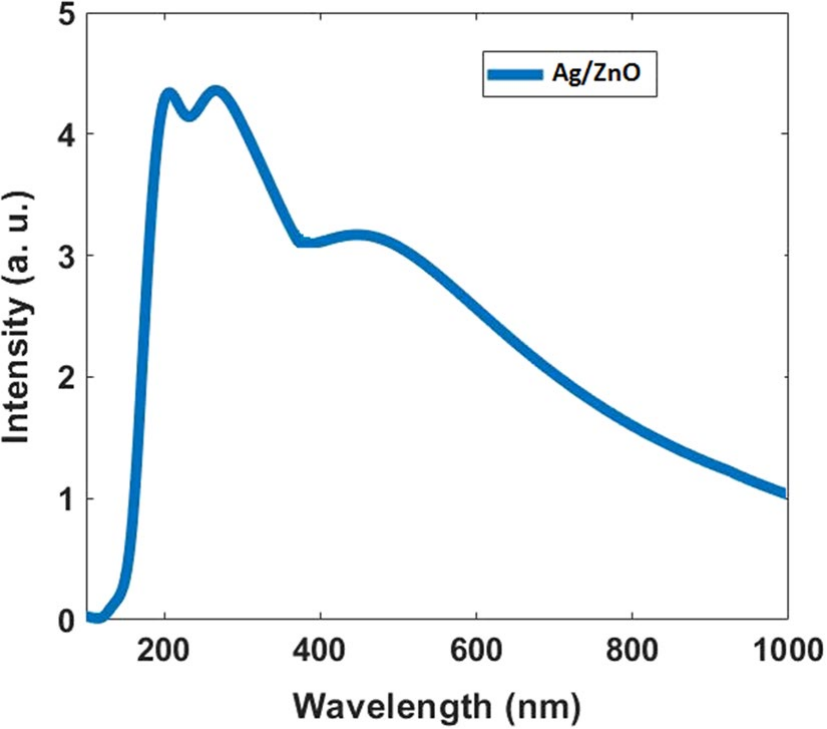
Absorption spectrum for synthesized Ag/ZnO Nanoparticles.

**Figure 4:**
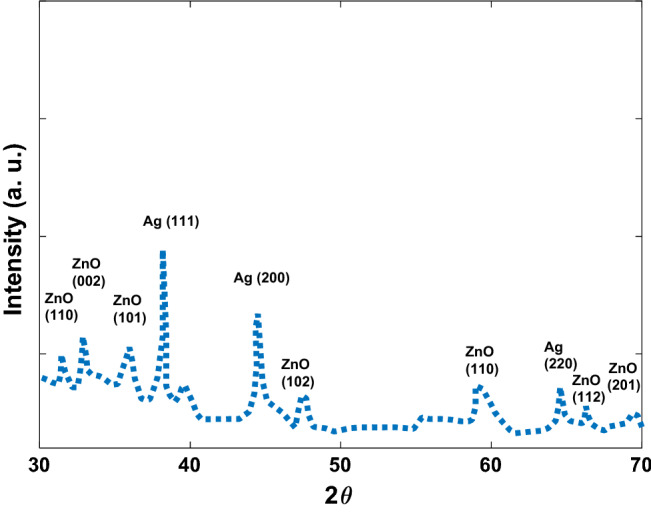
The XRD pattern of the synthesized NPs.

Figure 3 shows the absorption spectrum of Ag/ZnO synthesized nanoparticles. As the figure shows the synthesized materials have absorption peaks at 200, 250, and 450 nm. However, the bands are relatively broad and it covers all ranges of the UV-B and UV-Aspectra. This means using it on the skin can act as a sunscreen. The colloid dried at 80 °C for recording the XRD diffraction pattern (Fig. 4). As the Figure shows, all the diffraction peaks correspond to ZnO (JCPDS Card No. 36-1451) and Ag (JCPDS file No. 04-0783) NPs with low crystallinity.

**Figure 5:**
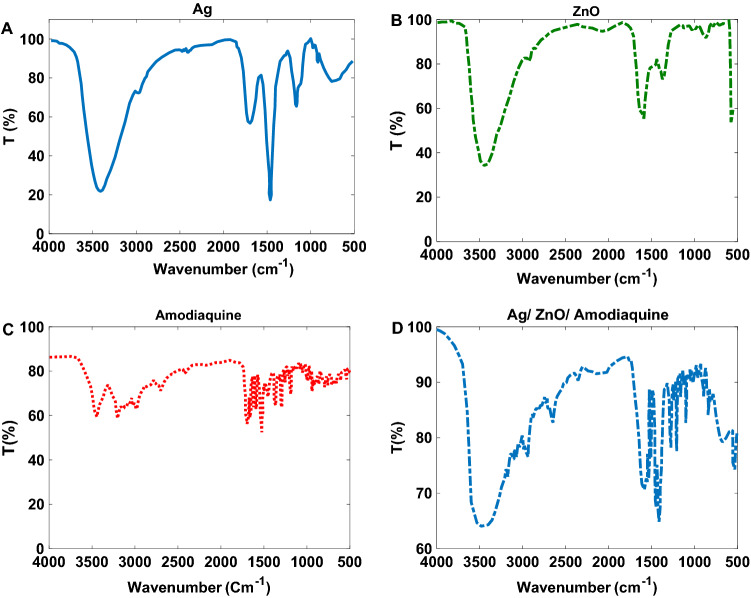
FT-IR spectra of the (**A**) Ag NPs (**B**) ZnO NPs, (**C**) Amodiaquine, (**D**) synthesized Ag/ZnO/ Amodiaquine NPs.

FT-IR spectra related to ZnO NPs, Ag NPs, Amodiaquine, and the synthesized NPs are shown in Fig. 5. FT-IR spectrum of the synthesized NPs shows existing of Ag, ZnO, and Amodiaquine absorption bands.

### Irritation and toxicity tests

**Figure 6:**
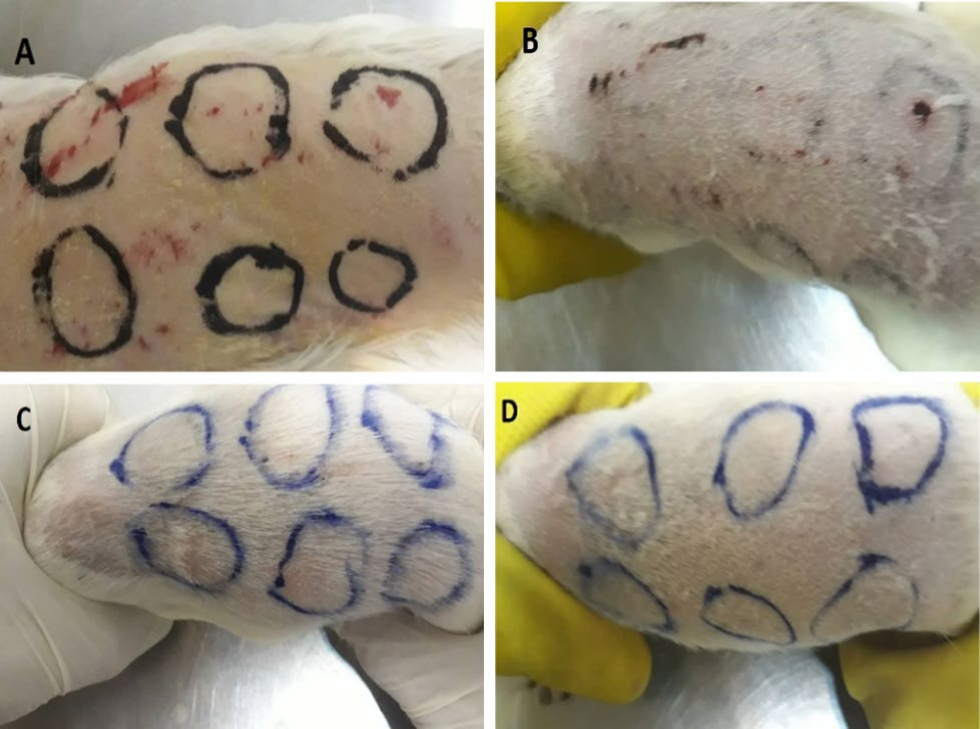
test irritation (**A**) the first day after removing the Fur (without exposure with colloid) (**B**) 1 weak after exposure with the synthesized colloid (**C**) 2 weeks after exposure (**D**) 4 weeks after exposure.

0.5 ml of the test substance was applied on the intact skin of 4 × 3 (three separate occasion test) Hindi pigs, in the dorsal region on the right and left sides (See Fig. 6). The back’s right caudal and left cranial area of 4 tested animals has been treated with the sample, while the left caudal area and right cranial area of the back has been used as control, treated with the inert normal saline only.

The application lasted for 4 h for a simple irritation test and 3 days for a repeated irritation test. The skin reactions were evaluated 1, 24, 48, and 72 h after the beginning of the treatment. No edema or erythema was observed in all animals treated with the test material. The positive control group shows edema erythema as mentioned in Table 1. Based on the results, interpreted according to BS EN ISO 10993-10; 2016, the test substance did not cause any irritant effects after 24, 48, and 72 h on the skin. Figure 4 shows guinea pigs used for the irritation test of the synthesized nanoparticles.

**Table 1:** Edema or erythema tests for 4, 24, 48, and 72 h.

Animal No	Positive Control	Negative Control	Observation + 24 h	Observation + 48 h	Observation + 72 h
1	Er ++++ Ed ++++	Er − Ed −	Er − Ed −	Er − Ed −	Er − Ed −
2	Er ++++ Ed ++++	Er − Ed −	Er − Ed −	Er − Ed −	Er − Ed −
3	Er ++++ Ed ++++	Er − Ed −	Er − Ed −	Er − Ed −	Er − Ed −
4	Er ++++ Ed ++++	Er − Ed −	Er − Ed −	Er − Ed −	Er − Ed −

*Er* erythema, *Ed* edema.

80 µg/ml of Ag with 50 µg/ml ZnO dose levels showed no significant changes in behavior before and after the dermal administration of the synthesized material. Table 2 shows the general cage-side observations for the parameters studied.

**Table 2:** Cage-side observations for all animals’ parameters cage-side observation.

No	Parameters	Cage side observation
1	Condition of the fur	Normal
2	Skin	Normal
3	Subcutaneous swellings	Nil
4	Abdominal distension	Nil
5	Eyes—dullness	Nil
6	Eyes—opacities	Nil
7	Pupil diameter	Normal
8	Ptosis	Nil
9	Color and consistency of the feces	Normal
10	Wetness or soiling of the perineum	Nil
11	Condition of teeth	Normal
12	Breathing abnormalities	Nil
13	Gait	Normal

Bodyweight is an important factor to monitor the health of an animal. Loss in body weight is frequently the first indicator of the onset of an adverse effect. A dose, which causes a 10% or more reduction in body weight, is considered to be a toxic dose^65^. It is considered the dose, which produces a minimum toxic effect, irrespective of whether or not it is accompanied by any other changes. All the animals from the treated groups did not show any significant decrease in body weights for all 14 days as compared with the 0- day values. There was no significant change in food and water intake of the test animals at all dose levels for all days.

From the results of this study, it is observed that there is no change in body weight, food and water consumption by the animals from all dose groups, and there was no mortality recorded even at the highest surface area level i.e. 100% surface area, which proves that the synthesized nanomaterial have no significant toxic effect in Hindi pigs through the dermal application. So, the synthesized drug has been considered practically nontoxic from this route of administration.

The toxicity of the synthesized material was tested based on the Draize eye protocol on the eyes of white rabbits and no biochemical, behavioral, or pathological changes were observed during 2 months. The Draize rabbit eye test is an acute toxicity test for assessing the effects of chemicals, substances, and mixtures in terms of their potential to cause irritancy or damage to the body cells.

### Mechanism of Antiviral effects

Metals and metal ions tend to bind to O, N, and S-ligand atoms through interactions that are often strong and selective. These interactions are based on coordination chemistry.

Hard-soft acid-base theory (HSAB theory) is another concept that plays a fundamental role in the reactivity of metals. The HSAB classification, which has been determined empirically, provides an arrangement of transition metals according to their preferences for specific organic ligands. For example, soft acids (such as Hg(II), Cu(I), Ag(I), and Cd(II)) and borderline acids (such as Co(II), Ni(II), Cu (II) and Zn(II)) tend to bond tightly with soft bases, such as sulfhydryl groups (RSH) found in proteins^66^. As a result, the antibacterial toxicity of these metals is roughly proportional to their affinity for S atoms. The Covid-19 virus has spike glycoprotein (S), membrane (M), envelope (E), and nucleocapsid (N) proteins, in its structure. Surface proteins have sulfhydryl groups And Ag and Zn atoms can inhibit viral infection by blocking the spike protein and angiotensin of the *SARS-CoV* which can lead to the formation of protein disulfides and the depletion of antioxidant reserves, particularly glutathione, within microbial cells. Also, in addition to the destruction of the active site, metal substitutions at non-catalytic metal-binding sites can inhibit enzyme activity. This means the synthesized Ag/ZnO NPs can bind to viral surface proteins having sulfhydryl groups and break sulfide bonds to disrupt the protein, leading to disruption of viral binding to the target cell receptor^67–70^ and poisoning the bacteria and virus cells. Also, a few papers reported that Aqueous ZnO suspensions have also been reported to increase the levels of reactive oxygen species (ROS), mainly hydroxyl radicals, which contribute to the antibacterial activity of ZnO nanoparticles^60^.

It can be assumed that the main mechanism of antiviral action of Ag/ZnO nanoparticles against *SARS-CoV-2* is preventing viral binding or interfering with virus entry or by damaging surface proteins, especially through the reaction of Amodiaquine molecules with membrane proteins and disrupting the structural integrity of virions and inhibiting the entry stage of the virus. In addition, these nanoparticles can enter the cellular cytoplasm and interact with the nucleic acids and disrupt the performance of the virus and possibly inhibit viral infection from infected cells to non-infected cells^71^. Further studies are needed to further investigate the antiviral performance of the synthesized nanoparticles on *SARS*-*CoV* for in-depth clarification.

### Antibacterial activity of Synthesized nanoparticles

Table 3 shows the effect of synthesized nanomaterials on examined bacteria. As the results show, the growth of all bacteria was inhibited completely after incubation with the synthesized nanoparticles at 15 μg/L (Ag)/50 µg/ml (ZnO), and the logarithmic reduction in bacteria number is more than 8 within 60 s (nearly 100%) which acceptable number is more than 5.

**Table 3:** The effect of the synthesized nanomaterials on examined bacteria.

Sample	Bacteria	Bacteria number in the first solution	60 s	120 s	Reference number
15 μg/L (Ag)/50 µg/ml (ZnO) of the synthesized Ag/ZnO	*E. coli*	1.2 × 10^9^	176	120	EN1276
	*Staphylococcus aureus*	5.3 × 10^9^	105	105	EN1276
	*Enterococcus hirae*	4.8 × 10^9^	120	110	EN1276
	*Pseudomonas aeruginosa*	1.3 × 10^9^	360	120	EN1276
	*Methicillin-resistant Staphylococcus aureus (MRSA)*	5.1 × 10^9^	211	112	EN1276

**Figure 7:**
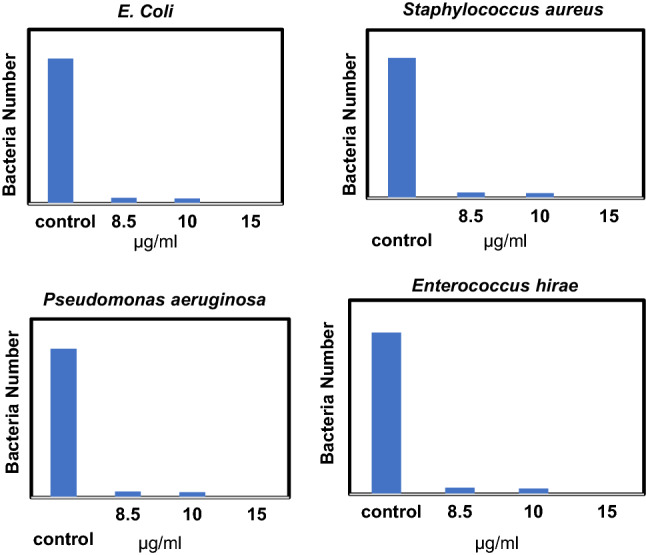
Effect of synthesized nanoparticles on *E. coli*, *Staphylococcus aureus*, *Enterococcus hira*, and *Pseudomonas aeruginosa*, in different concentrations (the concentrations of 8.5, 10 and, 15 µg/mL are related to Ag nanoparticles and the concentrations of ZnO NPs is 50 µg/mL for all).

The results for the effect of the synthesized nanomaterial on *E. coli* bacteria in the concentrations of 8.5, 10 and 15 µg/mL Ag with 50 µg/mL ZnO are shown in Fig. 7. The results indicate the antibacterial purpose can not be satisfied by concentrations less than 15 µg/mL with 50 µg/mL ZnO. The comparison of the obtained results with other reported nanoparticles indicates a much higher affectivity of the synthesized nanomaterials^72^. For example, the percentage of antimicrobial activity at 0.1, 1, 10, 25, and 50 mg/L for MnS/Ag_2_WO_4_ on *E. coli* has been reported as 66%, 78%, 89%, 99%, and 99.9%. The percentage of antimicrobial activity at 0.1, 1, 10, 25, and 50 mg/L for MnS on *E. coli* and *B. subtilis* are 23%, 41%, 66%, 74%, and 77% and 15%, 22%, 39%, 63%, and 74% respectively. The percentage of antimicrobial activity at 0.1, 1, 10, 25, and 50 mg/L for Ag_2_WO_4_ on *E. coli* and *B. subtilis* are 35%, 52%, 63%, 79% and 93% and 25%, 42%, 60%, 72%, and 85% respectively^73^.

### Antifungal activity of Synthesized nanoparticles

Table 4 shows the effect of synthesized nanomaterials on examined fungi. As the results show, the growth of all fungi was inhibited completely after incubation with the synthesized nanoparticles at 15 μg/L (Ag)/50 µg/ml (ZnO), and the logarithmic reduction in fungi number is more than 6 in 60 s (the acceptable reducing is more than 4). This is more than 8.7 in 120 s.

**Table 4:** The effect of synthesized nanomaterials on examined fungi.

Sample	Fungi	Fungi number at the first solution	60 s	120 s	Reference number
15 μg/L (Ag)/50 µg/ml (ZnO) of the synthesized Ag/ZnO	*Candida albicans*	6.1 × 10^9^	165	110	EN1650
	*Aspergillus*	6.2 × 10^9^	120	96	EN1650

### Antiviral activity of Synthesized nanoparticles

Table 5 shows the effect of synthesized nanomaterials on examined viruses. As the results show, the growth of the *herpes simplex* virus* (*HSV*)* was inhibited with the synthesized nanoparticles at 15 μg/L (Ag)/50 µg/ml (ZnO), and the logarithmic reduction in virus number is more than 6 in 30 s which an acceptable number is more than 4. This reduction is more than 7.1 for *Covid 19*. And this means the produced medicine can be applied for inhibiting of *Covid 19* easily.

**Table 5:** The effect of synthesized nanomaterials on examined viruses.

Sample	Virus	Virus number at the first solution	30 s	Reference number
15 μg/L (Ag)/50 µg/ml (ZnO) of the synthesized Ag/ZnO	*Herpes simplex virus* (*HSV*)	1 × 10^8^	122	EN1052
	*Influenza (H1N1)*	1 × 10^6^	5	ISIRI16676
	*Covid 19 (SARS-COV-2)*	3.2 × 10^8^	21	EN14476

### Clinical trial

The medicine was tested as a mouthwash on 500 people, 280 of whom had symptoms of *Covid 19* and 220 who did not have symptoms but were caring for the patient. Of the 280 people who had symptoms, 28 had more than 70 percent lung involvement. People with mild symptoms all recovered completely after 9 gargles (three times a day). People with more symptoms recovered after 9 gargles with medication prescribed by a doctor. And people who did not have symptoms did not get sick despite being exposed to the virus.

## Conclusions

In this paper, Ag/ZnO/Amodiaquine nano colloid was synthesized and characterized. Antibacterial, antiviral, antifungal, and toxicity effects of synthesized nano colloid in different concentrations were investigated. As the results show, the synthesized nanomaterial with a concentration of 100 µg per milliliter did not show any toxicity. Antibacterial tests of this material showed that a concentration of 15 μg/L (Ag)/50 µg/ml (ZnO) is sufficient to reduce bacteria by more than 6 logs in 60 s. This concentration of synthesized colloids also shows very good antifungal results. The antiviral effects of the synthesized nano colloid were evaluated on H1N1, herpes simplex, and Covid-19 viruses. In all of these cases, the synthesized nanomaterials in less than 30 s show a significant reduction of the virus, which in the case of *Covid 19* is 7.1 log. The clinical results of the synthesized colloid as a mouthwash show promising effects in curing and preventing the progression of *Covid 19* disease.

## Supplementary Information

Supplementary Information 1.

Supplementary Information 2.

Supplementary Information 3.

## Data Availability

We provide information on the search routines used to locate, and then download, those records. Those instructions allow an interested party with a suitable license to those databases to regenerate comparable datasets.

## Abbreviations

WHO: Health Organization
NPs: Nanoparticles
PVP: Polyvinyl pyrrolidone
MRSA: Methicillin-resistant *Staphylococcus aureus*
HSV: HERPES simplex virus
