# Supplementary figures and images for: Strong anti-viral nano biocide based on Ag/ZnO modified by amodiaquine as an antibacterial and antiviral composite
Source: Sci Rep. 2022 Nov 19;12:19934. doi: 10.1038/s41598-022-24540-8 (PMC9675852; doi:10.1038/s41598-022-24540-8)

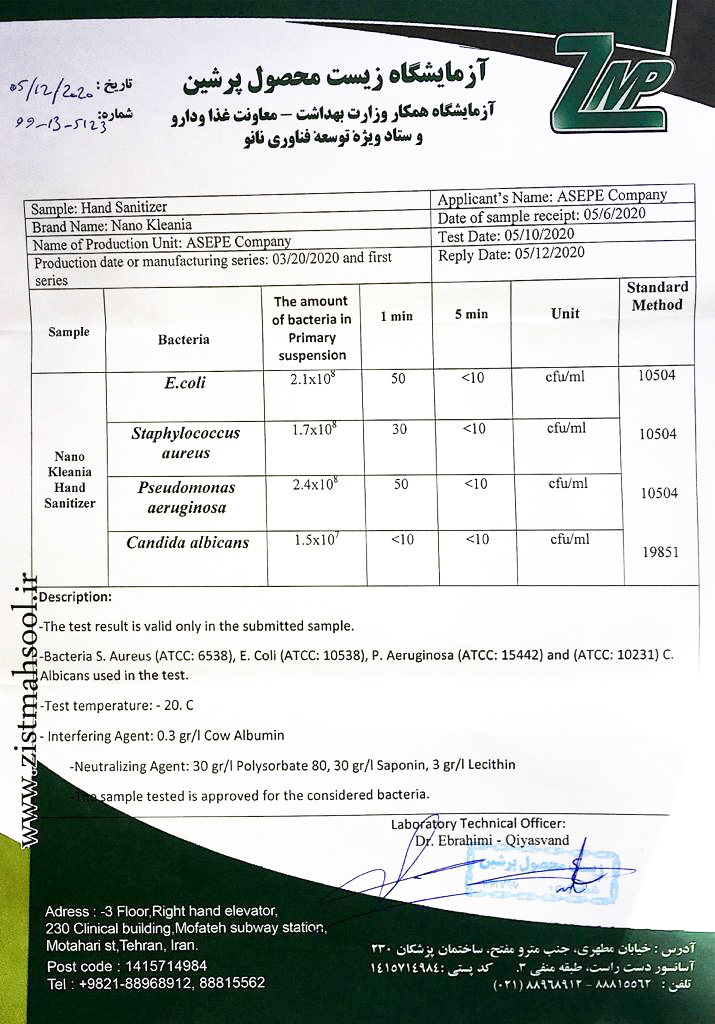

Supplement: Supplementary file 1 — Supplementary Information 1. [file 41598_2022_24540_MOESM1_ESM.jpg]
