# Supplementary material for: Strong anti-viral nano biocide based on Ag/ZnO modified by amodiaquine as an antibacterial and antiviral composite
Source: Sci Rep. 2022 Nov 19;12:19934. doi: 10.1038/s41598-022-24540-8 (PMC9675852; doi:10.1038/s41598-022-24540-8)
Supplement: Supplementary file 2 — Supplementary Information 2. [file 41598_2022_24540_MOESM2_ESM.pdf]

ANTİMİKROP	ANTİMİKROP ARGE VE BİYOSİDAL ANALİZ MERKEZİ	NAC
	MİKROBİYOLOJİK ANALİZ SONUÇ RAPORU	TESTING LABORATORY ISO/IEC 17025:2017 NAC-013-TL

RAPOR KAYIT NUMARASI	R-21-0250
RAPOR TARİHİ	11.08.2021
NUMUNE KAYIT NUMARASI	COV-21-0250
ÜRÜNÜN TAM ADI	NANO KLEANIA
ÜRÜN/RUHSAT SAHİBİ	HMC NATURAL TARIM VE BİTKİSEL ÜRÜNLER LTD.ŞTİ.
NUMUNE AKTİF MADDELERİ ve ORANLARI	-
NUMUNE FORMÜLASYON ŞEKLİ	Sıvı
NUMUNE AMBALAJ MALZEMESİNİN CİNSİ	Sprey şişe
NUMUNE GELİŞ TARİHİ	15.07.2021
NUMUNEYİ GÖNDEREN KURUM ADI	HMC NATURAL TARIM VE BİTKİSEL ÜRÜNLER LTD.ŞTİ.
NUMUNENİN GELİŞ SEBEBİ, MÜHÜR DURUMU VE MİKTARİ	BİYOSİDAL ANALİZ- MÜHÜRSÜZ- 100 ml
NUMUNENİN ÜRETİM YERİ ADRESİ	HMC NATURAL TARIM VE BİTKİSEL ÜRÜNLER LTD. ŞTİ. Malıköy Başkent OSB Mh. 2.Cd. No:8 Sincan/ANKARA
NUMUNE ÜRETİM VE SON KULLANMA TARİHİ	-
NUMUNE ŞARJ/SERİ NUMARASI	-
ANALİZ BAŞLANGIÇ VE BİTİŞ TARİHİ	31.07.2021-04.08.2021
ANALİZ METODU	Virüsidal Analiz
ANALİZ SONUÇLARI	Ek-1/PR-13-FR-45-03 Virüsidal Test Sonuç Formu sunulmuştur.

Tarih: 11.08.2021
Birim Sorumlusu
Uzman Biyolog
Fulya PAK

Tarih: 11.08.2021
Raporu Hazırlayan
Gamze DİNCER CENGİZ

Tarih: 11.08.2021
Sorumlu Yönetici
Sorumlu Yönetici
Prof. Dr. Murat ERTÜRK

ANTİMİKROP ANTİMİKROBİYAL MAD. LAB AR-GE MÜH. VE DAN. HİZ.KİMYA SAN. TİC.LTD.ŞTİ.
Nasuh Akar Mah. Süleyman Hacıabdullahoğlu Cad. No:3 Çankaya / ANKARA
Hitit V.D.No:070 031 [illegible] Mersis No:[illegible]

№ 21250

ANTİMİKROP	ANTİMİKROP ARGE VE BİYOSİDAL ANALİZ MERKEZİ<br>MİKROBİYOLOJİK ANALİZ SONUÇ RAPORU	NAC TESTING LABORATORY ISO/IEC 17025:2017 NAC 033-TL

EK 1: SONUÇLAR

ANALİZ TÜRÜ VE YÖNTEM	KULLANILAN MİKROORGANİZMA	SONUÇ	BAKTERİSİDAL ETKİ (% AZALMA)	TEMAS SÜRESİ
BAKTERİSİDAL ANALİZ	*P. aeruginosa*	-	-	-
	E. hirae	-	-	-
	S. aureus	-	-	-
	E. coli	-	-	-
	E. coli K12	-	-	-
	B. subtilis	-	-	-

	KULLANILAN MİKROORGANİZMA	SONUÇ	VİRÜSİDAL ETKİ (% AZALMA)	TEMAS SÜRESİ
VİRÜSİDAL ANALİZ	*Poliovirüs Tip 1 (LSc 2ab suşu)**	-	-	-
	*Adenovirüs Tip 5 (Adenoid 75 suşu)**	-	-	-
	*M. Norovirus (S99 Berlin suşu)**	-	-	-
	COVID-19 (SARS-COV-2) (Klinik İzolat) (GenBank=MT955161.1)	7,17 LOG	>%99,99	120 Saniye

	KULLANILAN MİKROORGANİZMA	SONUÇ	FUNGUSİDAL ETKİ (% AZALMA)	TEMAS SÜRESİ
FUNGİSİDAL ANALİZ	*C. albicans*	-	-	-
	A. brasiliensis	-	-	-

ANALİZ YÖNTEMİNİN ÖZETİ	TS EN 14476+A2 standartının gerektirdiği koşullara göre analiz yapılmıştır. Buna göre NANO KLEANIA isimli ürün kullanıma hazır şekilde deneye alındığında kirli şartlarda (3,0 g/l bovine albumin solüsyonu) ve oda ısısında 120 saniyede muamele edilerek *COVID-19 (SARS-COV-2) (Klinik İzolat) (GenBank=MT955161.1)* deney organizması üzerindeki virüsidal etkisi analiz edilmiştir.

Bu rapor, laboratuvarın yazılı izni olmadan kısmen kopyalanıp çoğaltılamaz, ilgili kurumlar harici kullanılamaz.
İmzasız raporlar geçersizdir.
Bu rapor sadece yukarıda bilgileri geçen (laboratuvarımıza ulaştırılan) numune için geçerlidir.
Laboratuvarımız numune alma işlemini gerçekleştirmemekte olup, numune alımından kaynaklanan hatalar veya numunenin bütünü temsil etmemesinden dolayı oluşacak sorunlardan laboratuvarımız sorumlu değildir.
Bu rapor, reklam amaçlı kullanılamaz.
(*) işaretli deneyler akreditasyon kapsamı dahilindedir.

ANTİMİKROP ANTİMİKROBİYAL BİYOSİDAL LAB. AR-GE MÜH. VE DAN. HİZ. KİMYA SAN. TİC. LTD. ŞTİ.
Nasuh Akar Mah. Süleyman Hacıabdullahoğlu Cad. No 37/[illegible]
Çankaya / ANKARA
Hitit V.D. No: 070 031 8715 Mersis No: 0070031871500[illegible]

№ 21250

ANTİMİKROP AR-GE VE BİYOSİDAL ANALİZ MERKEZİ

TEST SONUÇLARI (Virüsidal Test)

**Test Adı:**	COV-21-0250 kodlu ürünün TS EN 14476+A2 standartına göre virüs öldürme etkinliğinin değerlendirilmesi		
**Ürün İsmi:**	NANO KLEANIA	**Ürün Kodu:**	COV-21-0250
**Test Standardı:**	TS EN 14476+A2		
**Test Tarihi:**	31.07-04.08.2021		
Çalışma Şartları	**Kullanım şekli**	Kullanıma Hazır	
	**Sulandırıcı**	-	
	**Test yoğunluğu**	%80 Konsantrasyon	
	**Sulandırıldığında görünüm**	-	
	**Temas süresi**	120 saniye	
	**Test ısısı**	22-24^{0}C	
	**Engelleyici**	Kirli Şartlar: 3,0 g/l bovine albumin solüsyonu+Eritrosit	
	**Test maddesi görünümü**	-	
	**İnkübasyon ısısı**	37 ^{0}C	
	**Nötralizasyon Yöntem**	Dilüsyon Nötralizasyon	
	**Test Organizması**	*COVID-19 (SARS-CoV-2) (Klinik İzolat) (GenBank=MT955161.1)*	
	**Test Hücresi**	VERO E6 hücre hattı	
**Testi Yapan Kişi**	**Adı/Soyadı: Murat ERTÜRK**	**İmza:**	

Şekil 1. **NANO KLEANIA 'nın kirli şartlarda *COVID-19 (SARS-CoV-2) (Klinik İzolat) (GenBank=MT955161.1) virüsü*ne karşı virüsidal etkisi**

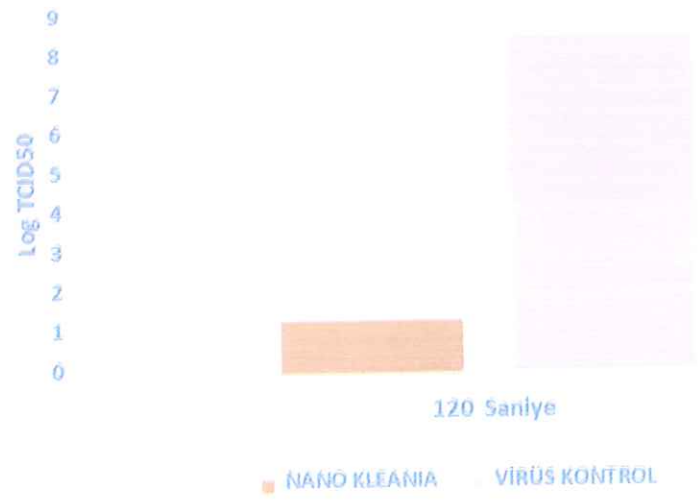

ANTİMİKROP ANTİMİKROBİYAL MAD. LAB. AR-GE MÜH. VE DAN. HİZ. KİMYA SAN. TİC. LTD. ŞTİ
Nasuh Akar Mah. Süleyman Hacıabdullahoğlu Cad. No:[illegible]
Çankaya / ANKARA
[illegible]

№ 21250

Test Maddesi	Test Yoğunluk	Engelleyici	Sitotoksisite (Log TCID50)	.......saniye sonra Log TCID50					120 saniye sonra >4 Log
				0	30	60	120	150	
NANO KLEANIA	%80	Temiz Şartlar (3 g/L BSA)	-	-	-	-	-	-	-
		Kirli Şartlar (3 g/L BSA+Eritrosit)	0,5	-	-	-	1,33	-	7,17
VİRÜS KONTROL	%80	Temiz Şartlar (3 g/L BSA)	-	-	-	-	-	-	-
		Kirli Şartlar (3 g/L BSA+Eritrosit)	-	8,50	-	-	8,50	-	-

* Log azalma= Log Virus Kontrol t120 saniye– Log Test Maddesi t120 saniye

Test Maddesi	Yoğunluk	Engelleyici	Temas Süresi (Saniye)	Sulandırma (Log)*						
				-2	-3	-4	-5	-6	-7	-8
NANO KLEANIA	%80	Kirli Şartlar (3,0 g/L BSA)	120	444<br>440	000<br>000	000<br>000	000<br>000	000<br>000	000<br>000	000<br>000
NANO KLEANIA Sitotoksisite	%80	Kirli Şartlar (3,0 g/L BSA)	-	000<br>000	000<br>000	000<br>000	000<br>000	000<br>000	000<br>000	000<br>000
Formaldehit	% 0.7	PBS	-	-	-	-	-	-	-	-
			-	-	-	-	-	-	-	-
Formaldehit Sitotoksisite	% 0.7	PBS	-	-	-	-	-	-	-	-
Virüs Kontrol	-	Kirli Şartlar (3,0 g/L BSA)	0	444<br>444	444<br>444	444<br>444	444<br>444	444<br>444	241<br>132	000<br>000
			120	444<br>444	444<br>444	444<br>444	444<br>444	444<br>444	444<br>444	000<br>000

* 0-4 sayıları virüsün sebep olduğu cpe varlığını ve kuyucuk içeriği hücre tabakasındaki cpe oranını ifade eder (0: cpe yok; 1: % 25 cpe; 2: % 50 cpe, 3: % 75 cpe; 4: % 100 cpe).

ANTİMİKROP ANTİMİKROBİYAL MAD. LAB. [illegible] N. HİZ. KİM. [illegible] TİC. LTD. ŞTİ.
Nasuh Akar Mah. Süleyman Hacıabdullahoğlu Cad. No:37
Çankaya / ANKARA
Hitit V.D. No:070 031 8715 Mersis No:007003187150[illegible]

№ 21250

SONUÇ

Yöntem doğrulaması gerçekleştirilen test ancak aşağıdaki doğrulama şartlarını sağladığında geçerlidir.

Şartlar	*Yorum*
1.1. Virus test süspansiyonu, virus titresinde 4 log fark göstermelidir.	*Virus titresi 4 log fark göstermeye yeterlidir.*
1.2. Ürünün sitotoksisitesi, virus titresinde 4 log fark göstermeye engel olmamalıdır.	*Ürün toksisitesi 4 log fark göstermeye engel değildir*
1.3. İnterferans kontrol test sonucu virus kontrol ile karşılaştırıldığında virus titresinde 1 Log'dan fazla fark göstermemelidir.	*İnterferans kontrol uygundur.*
1.4. Aktivite baskılama test sonucunda virus titre farkı ≤ 0,5 log olmalıdır.	*Buz soğuk medyum ile sulandırma+ kromatografi sonrası elde edilen eluat virus üremesine engel değildir.*
1.5. Referans inaktivatörün > 4 log virusidal etkisi > 60 dakika testinde uygun olmalıdır.	*Referans inaktivatör olarak kullanılan Formaldehit > 4 log virusidal etkisini > 60 dakikada göstermiştir.*

TS EN 14476+A2 standardına göre, ***NANO KLEANIA*** *isimli ürün kullanıma hazır şekilde deneye alındığında kirli şartlarda, oda ısısında 120 saniyede COVID-19 (SARS-CoV-2) (Klinik İzolat) (GenBank=MT955161.1) virüsüne karşı VİRUSİDAL ETKİLİDİR.*

TARİH:11.08.2021
ANKARA

Mikrobiyolojik Analiz Laboratuvar Birim Sorumlusu	*Kalite Sorumlusu*	*Sorumlu Yönetici*
Uzman Biyolog Fulya PAK	Ceren ÖZKAN	Sorumlu Yönetici Prof. Dr. Murat ERTÜRK

ANTİMİKROP ANTİMİKROBİYAL ARAŞ. LAB. AR-GE MÜH. VE DAN. HİZ. KİMYASAN TİC. LTD. ŞTİ
Nasuh Akar Mah. Süleyman Hacıabdullahoğlu Cad. No 37-
Çankaya / ANKARA
Hitit V.D.No:070 031 8715 Mersis No:0070031871500[illegible]

№ 21250
